# Sperm DNA Fragmentation: causes, evaluation and management in male infertility

**DOI:** 10.5935/1518-0557.20230076

**Published:** 2024

**Authors:** Syed Waseem Andrabi, Anam Ara, Ankur Saharan, Mir Jaffar, Nivita Gugnani, Sandro C. Esteves

**Affiliations:** 1 Nova Southend Fertility and IVF centre, New Delhi; 2 University of Manitoba, Winnipeg, Canada; 3 Amity University, Lucknow, India; 4 Valley Fertility Centre, Srinagar, India; 5 Ridge IVF Centre, New Delhi; 6 ANDROFERT, Andrology and Human Reproduction Clinic, Campinas, SP, Brazil; 7 Department of Surgery (Division of Urology), University of Campinas (UNICAMP), Campinas, SP, Brazil; 8 Faculty of Health, Aarhus University, Aarhus, Denmark

**Keywords:** reproductive biology, male infertility, sperm, DNA fragmentation, assisted reproductive techniques

## Abstract

Male infertility is a great matter of concern as out of 15% of infertile couples in the reproductive age, about 40% are contributed by male factors alone. For DNA condensation during spermatogenesis, constrained DNA nicking is required, which if increased beyond certain level results in infertility in men. High sperm DNA Fragmentation (SDF) majorly contributes to male infertility and its association with regards to poor natural conception and assisted reproductive technology (ART) outcomes is equivocal. Apoptosis, protamination failure and the excess of reactive oxygen species (ROS) are considered to be the main causes of SDF. It’s testing came into existence because of the limitations of the conventional methods in explaining infertility in normozoospermic infertile individuals. Over the past 25 years, SDF’s several testing strategies have been proposed to diagnose the aetiology of infertility. Various treatments combined with sperm selection techniques are being used alone or in combination to reduce DNA fragmentation index (DFI) and obtain spermatozoa with high quality chromatin for assisted reproduction. This review summarises SDF’s main causes, its impact on fertility and clinical outcomes in assisted reproduction, the need to perform test, testing procedures, and the treatment strategies.

## INTRODUCTION

Couples failing to conceive after one year of unprotected coitus are considered to be infertile. Infertility affects about 15% population of the reproductive age, of which male factors contribute to about 50% of the cases. Various factors associated with male infertility include anatomical abnormalities, oxidative stress, varicocele, an endocrine disorder, systemic disease and infection (Panner Selvam & Agarwal, 2018). A major development in ART to overcome male infertility issues, was the advent of intracytoplasmic sperm injection (ICSI); however, this development has also led to a major ignorance of the aetiology of male factor infertility, probably one big reason why ART results (live birth rates) are static around 40% only (Rilcheva *et al*., 2016). These results suggest that the male factor in ART clinics needs to be distinguished and understood better at the molecular level, which may increase live birth rates in ART. Male infertility is driven by various factors like hormonal regulation and genetic/epigenetic alterations. Cooper *et al*. (2010) elaborated conventional semen analysis to be a crucial diagnosis method for male infertility factors all across the world. However, the WHO parameters cannot explain infertility, due to its reference being subjected to only poor semen parameters. The applicability of the conventional semen analysis is still lacking in infertile males as 40% of infertile men show semen parameters within the WHO normal reference range (Agarwal *et al*., 2020). Thus, the conventional semen analysis alone cannot help in analysing various molecular subcellular factors associated with male factor infertility.

**Figure 1:**
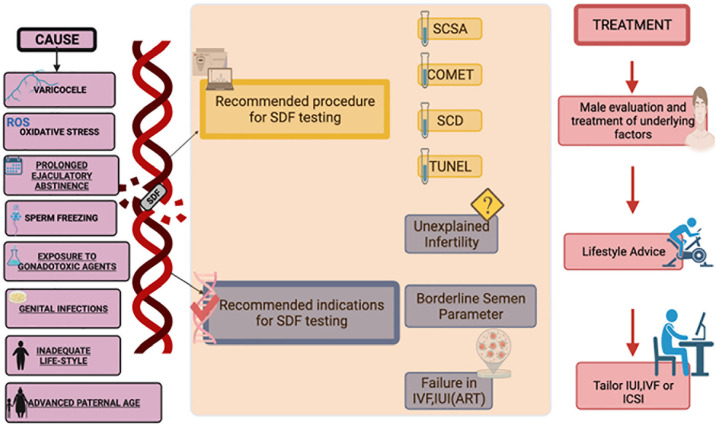
SDF causes, tests and treatments (Abbreviations used: SCSA-Sperm chromatin structure assay, SCD-Sperm Chromatin Dispersion IUI-Intrauterine Insemination, IVF-In Vitro Fertilization, ICSI-Intracytoplasmic Sperm Injection).

Sperm DNA fragmentation has been reported to be associated with male infertility and reproductive failure (Panner Selvam & Agarwal, 2018). During the late stage of spermatogenesis, chromatin condensation and packaging need events like Lysine rich histone substitution by arginine-rich protamines and disulphide bond formation in cysteine residues for the appropriate packaging and chromatin protection from any chemical and natural injury. A significant rise in the level of SDF interferes with chromatin condensation, resulting in sperm DNA abnormality (Agarwal *et al*., 2020). It has been reported that infertile men have high level of SDF in spermatozoa. DFI was found to be significantly higher in oligozoospermic males compared to normozoospermic males and no successful pregnancy was observed in embryos derived from high DFI sperm samples (Esteves *et al*. 2015a). In order to differentiate fertile and infertile men on the basis SDF level, a meta-analysis on 4000 men from 27 studies concluded that SDF threshold that can differentiate between fertile and infertile men was 20% (Roque & Esteves, 2018). Many conditions such as varicocele, reactive oxygen species, lifestyle, genital infections, advanced paternal age, chemo-therapeutic drug of testicular cancer (bleomycin etoposide), radiotherapy, urogenital infection, and cigarettes have been associated with elevated SDF levels (Osman *et al*., 2015; González-Marín *et al*., 2012; Esteves *et al*., 2021; Zakaria *et al*., 2021). SDF also results in impaired fertilization, sub-optimal embryo quality, reduced pregnancy rate and increased abortion during Invitro fertilization (IVF). Similarly, it interfered more by negatively affecting fertilization rate, clinical pregnancy and live birth rate (Zhao *et al*., 2014; Osman *et al*., 2015; Arafa *et al*., 2021) when ICSI was performed. In a study, cumulative live birth rate was lowered in patients undergoing IVF due to high SDF, further embryo morphokinetic nature like delayed cell cleavage and blastulation was also affected by high SDF (Malić Vončina *et al*., 2021; Setti *et al.*, 2021). Another study negatively correlated SDF with blastulation and pregnancy rate as high level of DFI promoted embryo arrest by inducing apoptosis (Simon *et al*., 2014). Thus, SDF needs to be considered as one of the important diagnostic tools to assess male infertility (Agarwal *et al*., 2020) (Figure 1).

In order to find a probable relationship between sperm DNA integrity and male infertility, many changes and adaptations have been brought up in sperm function tests, including advanced and stronger diagnosis tools (Cho *et al*., 2017). Apart from the conventional semen analysis tests, SDF can also be used in sperm function tests for the identification of better fertility outcomes in couples with unexplained infertility. Two guidelines were given by Agarwal *et al*. (2020) and Esteves *et al.* (2021) with respect to the SDF testing in which they have discussed about when to do SDF testing, how to test and how to treat. Both the guidelines recommended SDF testing, in cases of unexplained infertility, varicocele, RPL (recurrent pregnancy loss), failed but unexplained Intra-Uterine Insemination (IUI), IVF and ICSI and in patients exposed to lifestyle risk factors and environmental toxicants (Agarwal *et al*., 2020; Esteves *et al*., 2021). Other international societies such as American Urological Association (AUA) and American Society of Reproductive Medicine (ASRM) have recently published guidelines on male infertility and recommended that SDF testing is not preferred in the initial evaluation of male fertility, but should be done in patients with RPL (Schlegel *et al*., 2021a; 2021b). Similarly, the European Association of Urology (EAU) recommends SDF testing in patients with RPL or unexplained infertility (Tharakan *et al*., 2022). In this review article, we have discussed about the SDF causes, tests, its impact on male fertility, methods of prevention, and possible treatments.

## MATERIALS AND METHODS

### Literature search

**Figure 2:**
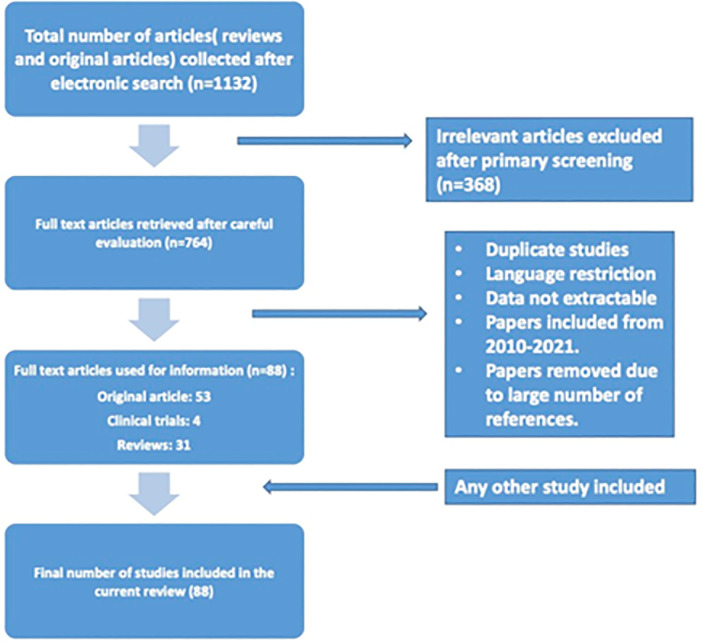
Flow chart illustrating the study selection criteria.

A thorough literature search was conducted across scientific databases Medline, PubMed, Cochrane review and Google Scholar to find articles and research. Keywords such as semen parameters, sperm DNA fragmentation, sperm DNA damage, male infertility, pregnancy, fertilization, Assisted Reproductive Techniques, IUI, IVF and ICSI were used in various combinations. The citations in each article were scanned to find other linked relevant research articles as shown in Figure 2.

### Screening

Out of the hits obtained upon literature search, we screened the abstracts of all articles from 2010-2021 to identify the studies on SDF in male infertility, methods to assess SDF, and the treatments aimed at alleviating SDF. Furthermore, we removed the duplicate articles from the hits. The full text of relevant articles were collected, followed by full text screening of all eligible articles, which was used to identify the final set of studies included for review.

### Analysis and presentation

The studies included in this review were classified in the following groups: those on the main causes of SDF, those assessing the impact of SDF on fertility and clinical outcomes in assisted reproduction, those evaluating the need to perform SDF tests, those detailing SDF testing procedures, and those presenting with the treatment strategies. Some of the studies fitted in more than one group.

The data presented in the studies in each group were analyzed together to draw conclusions about a specific question, which included the main causes and impact of of SDF on fertility and clinical outcomes in assisted reproduction. It also included the treatment methodology and popular SDF testing procedures.

### Causes of Sperm DNA Fragmentation

Sperm DNA is wound around histone proteins that are replaced by basic protamines for condensation during spermatogenesis, where torsional stress of double-strand DNA results in the nick, which later on is restored by appropriate reordering of chromatin. Somewhere, if these nicks aren’t repaired properly, it can result in DNA fragmentation and infertility in males. Different events that resulted in infertility due to sperm DNA damage in males include:

- Abnormal chromatin packaging and remodeling during spermatogenesis (Agarwal *et al*., 2016a).
- Apoptosis during epididymal sperm maturation (Gosálvez *et al*., 2015).
- Oxidative Stress (Zakaria *et al*., 2021)
- Varicocele, infections, inflammation of male genital tract, febrile illness, obesity, advanced age, environmental pollutants and toxins (Cho *et al*., 2017; Agarwal *et al*., 2020).
- Drugs, chemotherapy and radiotherapy (Esteves *et al*., 2015b; González-Marín *et al*., 2012)

SDF can be caused by different factors, which are divided into intrinsic and extrinsic categories. These categories are made after finding various processes involved in the SDF rise and in turn male subfertility.

### NSIC FACTORS

#### 1. Recombination deficiencies during spermatogenesis

The process of crossing-over results in recombination during meiosis can develop errors by creating DNA breaks via nucleases. Due to highly compact chromatin affinity for DNA-DNA and DNA-Protein cross-linking is more in sperm cells which makes it difficult for further condensation, examples of oestrogen found linked to DNA covalently in human spermatozoa and highly cross-linked chromatin in sperm (González-Marín *et al*., 2012).

#### 2. Abnormal Spermatid Maturation

For chromatin packaging, histone hyper-acetylation and breaks are important which are created as well as ligated by Topoisomerase II. In order to give relief to torsional stress that helps in the substitution of histone by protamines, DNA undergoes nicks (González-Marín *et al.,* 2012). Before epididymal transfer, chromatin packaging and DNA reinstatement are required but DNA fragmentation occurs due to incomplete sealing of temporary breaks that end up in incomplete sealing of DNA nicks in spermatozoa is indicative of defective chromatin remodelling during spermatogenesis (Panner Selvam *et al*., 2021).

#### 3. Protamine I and II Ratio

This substitution of histones by protamines, during the late stage of spermatogenesis, occurs for 85-95% of histone proteins in a step-wise series of events, including, hyperacetylation, substitution of transition protein and a final step in which histones are replaced by protamines I & II. PI and PII should be in a ratio of 1:1 for accurate packaging and gene expression (González-Marín *et al*., 2012). Alteration in protamines that are substituted in place of histone can also result in infertility. The variation from this ratio to a higher or lower value was observed in men having high levels of SDF (García-Peiró *et al*., 2011; Zeyad *et al*., 2018).

#### 4. Oxidative Stress

Subfertility in males is due to oxidative stress (OS) or ROS. The ROS formed is regulated by antioxidants found in semen. During oxidative stress, there is an imbalance between ROS and antioxidants (Zakaria *et al*., 2021).

The development of male infertility and sperm dysfunction due to ROS have been hypothesized and proven (Wright *et al*., 2014). Sperm binding to the zona pellucida is controlled by low level ROS just like superoxide anion radicals uplift acrosome reaction and capacitation. OS and reduction in antioxidant capacity of spermatozoa were found to be associated with rise in the ROS (Zakaria *et al*., 2021). Various causes of ROS include infection, inflammation, leucocytes, smoking, alcohol, radiation, toxic chemicals, diseases of the male reproductive accessory gland, genital tract inflammation, varicocele, testicular torsion or cryptorchidism (Wright *et al*., 2014).

ROS is generated in the sperm by two methods one is the nicotinamide adenine dinucleotide phosphate oxidase system (NADPH-oxidase) and another is the nicotinamide adenine dinucleotide-dependent oxido-reductase (NADH-oxidoreductase) at plasma-membrane and mitochondria respectively. It is furthermore elaborated below:

- The primary ROS generated in human spermatozoa is the superoxide anion(^.^O_2_^-^ ). This one-electron reduction product of O_2_ secondarily reacts with itself in a dismutation reaction, which is greatly accelerated by superoxide dismutase (SOD), to generate hydrogen peroxide (H_2_O_2_). In the presence of transition metals such as iron and copper, H_2_O_2_ and ^.^O_2_^-^ can interact to generate the extremely pernicious hydroxyl radical (^.^OH) (Haber-Weiss reaction) (Figure 3).
  **Figure 3:**
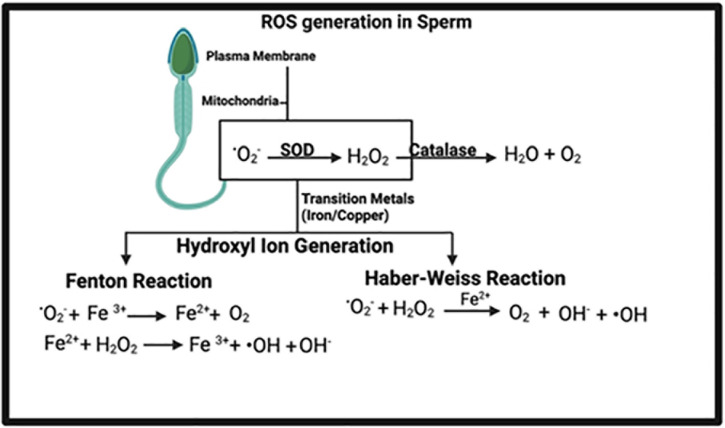
Equations illustrating the generation of ROS.
- Alternatively, the hydroxyl radical can be produced from hydrogen peroxide (Fenton reaction), which requires a reducing agent such as ascorbate or ferrous ions, as shown in the equation above. The hydroxyl radical is thought to be an extremely powerful initiator of the lipid peroxidation cascade (causing DNA fragmentation) and can precipitate loss of sperm functions.
- To catalyse the oxidation of H_2_O_2_and superoxide anion, GPX employs glutathione as an electron donor. In Sertoli cells, there is a significant amount of GPX. The head of the epididymis is where GPX is expressed, secreted, and detected in semen. GPX largely guards spermatozoa’s plasma membrane from lipid peroxidation.
- Peroxisomes contain the catalase enzyme (CAT), which breaks down H_2_O_2_ into H_2_O and O_2_. Although the amount of CAT in growing sperm is modest, the testicle always has a low degree of activity.

#### 5. Varicocele

Varicocele is one of the factors among 15% of male population and 40% among infertile men. Varicocele patients present with abnormal DNA and immature chromatin, leading to infertility; this was seen when Varicocele males were studied along with normozoospermic fertile males (Lira Neto *et al.,* 2021). One possible cause for poor semen quality and quantity along with increased DFI is scrotal hyperthermia or heat stress, causing oxidative stress in varicocele males (Gill *et al*., 2021). Varicocelectomy was found to be a possible treatment for males with varicocele as it can improve SDF levels, decrease in ROS, increase antioxidants and ultimately better ART outcomes. Varicocelectomy reduced SDF, improved sperm concentration, progressive motility, and morphology difference thus was considered as a treatment during abnormal DFI (Lira Neto *et al*., 2021).

#### 6. Genital Tract Infections

Reproductive tract infection is one of the common causes of male infertility, Urea plasma urealyticum, Chlamydia trachomatis, Mycoplasma, Staphylococcus aureus and Pseudomonas aeruginosa are types of organisms creating urogenital tract infection that may result in rising in SDF by interfering with sperm DNA/Chromatin structure (Agarwal *et al.,* 2020). These infections alter the protamine ratio in males as it was seen that protamine-I and protamine-II ratios were found to be altered in affected males along with high SDF levels (Zeyad *et al*., 2018). These infections can be treated by antibiotic therapy; however, it was found to trigger a further rise in the SDF level (González-Marín *et al*., 2012). It has been reported that genital tract infections increase ROS generation, which in turn increases oxidative stress induced DNA damage. Agarwal *et al*. (2018) found a positive correlation between chronic prostatitis and ROS generation.

#### 7. Age

Age has always been a factor that affects semen parameters negatively (Agarwal *et al*., 2020). ROS and lipid peroxidation increased with oxidative stress in mitochondria, which is related to aging (Agarwal *et al*., 2020; Pino *et al*., 2020). Age determining SDF level is not yet clear due to controversial reports, In one study on normozoospermic men, sperm DNA damage was higher (>30 % DFI) in older men (≥40 years) compared to younger males (<40 years) (Das *et al*., 2013). A positive correlation between the rising age and DFI and a negative correlation between the sperm parameters and DFI was reported (Hammiche *et al*., 2011). Evenson *et al*. (2020) conducted a study in 25,445 men attending infertility clinic and reported that the advanced paternal age is associated with increased SDF.

## EXTRINSIC FACTORS

### 1. Abstinence Period

As sperm accumulation occurs in the epididymis, ROS and Reactive Nitrogen species (RNS) generated by granulocytes can result in decreased sperm motility and DNA damage. Du Plessis et al., and his coworkers have reported the effect of H_2_O_2_ on sperm motility, ROS and nitric oxide level, which are the possible suspects of DNA damage (Wright *et al*., 2014). In another study conducted on the role of lower abstinence period, it was found that 3hr after the first ejaculation helps in managing SDF level for males with abnormal semen parameter, which can also increase the chances of pregnancy using ART (Dahan *et al*., 2021). In order to find the relation between the duration of abstinence and the percentage of DNA fragmentation during TUNEL study of 2458 men undergoing infertility investigation, a direct relationship was found between the abstinence period and the SDF levels (as the duration of abstinence increases, SDF increases as well) (Comar *et al*., 2017). A study on relation between ejaculatory abstinence (EA) and SDF was done that concluded EA of 1 and 2 days had least SDF (Agarwal *et al*., 2016b).

### 2. Lapse of Time from Ejaculation

Ejaculation time affects SDF levels gradually and is species dependent. In a study by Gosálvez *et al*., it was found that SDF and Protamine-IPI increases with an increase in expression of PII along with DNA stability increased with cysteine residue number. This report was generated from semen samples collected from 11 species, which was diluted, cryopreserved and thawed (Gosálvez *et al*., 2010).

### 3. Storage Temperature and Cryopreservation

For preservation and survival of sperm DNA, cryopreservation is one of the essential processes; however, it may change the structure and function of spermatozoa. A study found that sperm storage and processing methods have impact on SDF levels and hence it is necessary to consider this aspect when selecting a sperm for clinical use. Normozoospermic semen samples were found to have increased SDF index after cryopreservation (Le *et al*., 2019).

### 4. Other Factors

Heat stress, cytotoxic effect of radiotherapy and chemotherapy, position of the testis, antidepressant drugs give rise to abnormal SDF . Apart from that, various environmental and chemical factors also affect sperm DNA fragmentation. Factor like tobacco, whose consumption lead to the production of H_2_O_2_ through above mentioned fenton process leading to DNA damage. Similarly, elements like cadmium and lead, present in cigarette smoke can also cause DNA strand breaks. These elements were also found to be present in seminal fluid and are associated as an indicator of oxidative stress. Tobacco, which also contains nicotine, has been found to induce dsDNA break in sperm in-vitro, and its major primary metabolite form, cotinine, has been found in seminal vesicles of smoking males (Wright *et al*., 2014).

## IMPACT OF SDF ON CLINICAL OUTCOMES

### 1. SDF and Male Infertility

Although globally, fertility in males is decided by normal semen parameters; however, high SDF can can act as a barrier to male fertility (Agarwal *et al*., 2020). SDF levels are also affected by many factors like varicocele, obesity, unexplained infertility, idiopathic infertility, testicular cancer and ageing in men. SDF was categorized into viable and non-viable depending upon its impact on natural fertility in normozoospermic males, in viable SDF males, spermatozoa showed ability to fertilize, but later on failed in good embryo development, whereas non-viable SDF males are not able to fertilize (Muratori *et al*., 2020).

### 2. SDF and Natural Pregnancy Outcomes

The rate of natural pregnancy may ultimately be affected by DNA damage. The SDF index, when found to be around 20-30%, decreases the chance of natural pregnancy. The correlation between SDF and Pregnancy outcome was established by different studies, and was concluded that, more than 30% SDF level is critical for conception (Agarwal *et al*., 2016a). Recurrent spontaneous abortion was studied in 30 fertile couples and a negative correlation was found between SDF and sperm motility (Khadem *et al*., 2014). It has also been found that 30% of cases with SDF higher than 15% had recurrent miscarriages (Leach *et al*., 2015; McQueen *et al*., 2019).

### 3. SDF and Different ARTs (IUI, IVF, ICSI)

The inability to conceive was found to be increased in couples having SDF levels higher than 30% compared to couples with SDF levels lesser than 10 (Cho & Agarwal, 2017). Simon *et al*. (2014) stated 238 infertile samples where DNA damage was found to be associated with fertilizing ability during IVF (Table 1).

**Table 1:** Clinical impact of abnormal SDF level.

Clinical Scenario	Remarks	Study
Unexplained infertility	20% of men with unexplained infertility and 40-50% of men with idiopathic infertility have abnormal SDF.	Esteves *et al*., 2020 Gill et al., 2019
Recurrent Pregnancy loss or pregnancy loss	>30% SDF level was found critical for pregnancy outcomes. In-case of recurrent pregnancy loss SDF level was elevated after natural or assisted conception	Tan *et al*., 2019 Esteves *et al*., 2021
Failed IUI	Abnormal SDF negatively affect pregnancy rates in IUI	Sugihara *et al*., 2020
Failed IVF/ICSI	Abnormal SDF affect IVF/ICSI pregnancy rate. Men with high SDF on ICSI with testicular sperm gave better pregnancy result.	Esteves & Roque, 2019 Xie *et al*., 2020
Abnormal embryo development and birth Defects	SDF adversely affect embryo development. Icsi patient having high SDF had high aneuploidy and genomic abnormalities	Kim *et al*., 2019 Zheng *et al*., 2018

#### a. Intrauterine Insemination (IUI)

SDF has also been associated with altering IUI results. In one study conducted on 154 couples undergoing IUI in which 119 patients were not conceived as SDF was higher than 12% where as a study also confirmed a decrease in IUI pregnancy rate when SDF was more than 20% (Vandekerckhove *et al*., 2016; Chen *et al*., 2019; Agarwal *et al*., 2020). In another study, 30% SDF resulted in 13% rate of spontaneous abortion for 100 couples undergoing IUI cycles (Agarwal *et al.,* 2020). It was also found that the chance of conceiving gets as less as 3% in patients having SDF more than 30% in 387 IUI cycles among 637 couples (Wright *et al*., 2014). Thus, for better outcomes in IUI techniques, before starting treatment, couples should be advised to undergo SDF test to check DNA damage levels in advance. In support of this, another meta-analysis linked SDF and pregnancy rate during nine studies and 940 IUI cycles (Relative Risk (RR): 3.15; 95% Confidence Index (CI)9-: 1.46-6.79) (Sugihara *et al*., 2020).

#### b. In Vitro Fertilization (IVF)

SDF and IVF relationships are studied more thoroughly than IUI procedures in terms of pregnancy and miscarriage rates. In 210 couples undergoing IVF and ICSI procedures. an increase in failure to conceive by 1.3% was reported with a rise in 10% of SDF levels (Meseguer *et al*., 2011). Data recovered from 14 IVF/ICSI done including 2756 couples, elevated SDF resulted in miscarriage which was 10-15% but reached out to 23% in elevated SDF patients (Zhao *et al*., 2014; Esteves *et al*., 2021).

Lower DFI i.e., <2.7% was found to increase pregnancy rate in 20 articles reported by Zhang *et al.* (2015). Due to different designs and protocols having been used, result interpretation is the biggest challenge for all these research and reports. A report that stood against this relation was found in 550 Chinese couples in which 415 IVF and 135 ICSI patients showed no relation between SDF and pregnancy or live birth rates in IVF or ICSI (Xue *et al*., 2016). In a study, where 215 infertile couples undergoing ART and having fertilization problems, sperm DNA damage was associated with problems in fertilization, early embryonic development and pregnancy rate during IVF (Simon *et al*., 2014). A meta-analysis of 23 IVF/ICSI studies, including 6,771 cycles, corroborated these results. In this study, clinical pregnancy rates (23 studies; 6,771 cycles; RR: 1.57; 95% CI: 1.18, 2.09, *p*<0.01) and miscarriage rates (25 studies; 3,992 patients; RR: 0.85, 95% CI: 0.75-0.96, *p*<0.01) were negatively affected by the presence of elevated SDF (Deng *et al*., 2019).

#### c. Intracytoplasmic sperm injection (ICSI)

Sperm DNA has already been found associated with ICSI & IVF outcomes. This has been confirmed by different studies, where pregnancy outcomes of IUI, IVF and ICSI were studied and correlated with sperm DNA damage (Wright *et al*., 2014). It has also been found that pregnancy rate and embryo morphogenetic parameters depend on sperm DNA damage in ICSI patients. In the same study, it was concluded that pregnancy rates decrease with an increase in sperm DNA damage and developmental competence in blastocysts (more time will be required to reach blastocyst stage) (Wdowiak *et al*., 2015). In another study, where 269 couples undergoing ICSI, it was found that high SDF causes 2.2-fold rise in miscarriage rates (Robinson *et al*., 2012). The morphokinetic, cleavage and embryo quality was found to decrease when SDF is greater than 15% (Wang *et al*., 2022). Couples undergoing ART with male partners having low sperm DNA high birth rates (Osman *et al*., 2015). The study that stood against the role of SDF and clinical outcomes was a study conducted on 156 couples undergoing ART cycles (both IVF and ICSI), where no correlation was found between sperm damage and pregnancy rates (Anifandis *et al*., 2015). It was further suggested that failure in ICSI can be recovered by use of testicular sperm as might be due to decreased SDF along with other factors like oxidative stress in comparison to one in epididymal and ejaculated specimen giving higher reproductive success (Esteves *et al*., 2017; Pabuccu *et al*., 2017; Alharbi *et al*., 2020; Esteves & Roque, 2019; Xie *et al*., 2020).

### 4. SDF and Birth Defects

ART helps to overcome infertility in couples, however, the threat of transferring defective genomes to children can’t be identified as such (Table 1). However, Lewis & Simon (2010) in their study reported no transfer of defective genes to offspring in severe SDF treated males with ICSI procedure. However, multiple studies suggest either a direct or an indirect link between males with high sperm DNA damage and genetic abnormalities in offspring. Increased aneuploidy and genomic abnormalities were associated with an increase in SDF level undergoing ICSI treatment (Gharagozloo & Atiken, 2011). In mice, a study was reported where abnormality in offspring in the form of growth, behaviour, and increased prevalence of tumour were linked to increased SDF (Cho *et al*., 2017). Also, hereditary disorders, birth defects and nervous system dysfunction have been associated with aged males with disrupted DNA integrity (Kim, 2018).

## SDF TESTING WHEN?

In the past few years, significant amount of research has been conducted in relation to SDF and male infertility. Recently, clinical practice guidelines (CPG) included certain guidelines for SDF testing (Xie *et al*., 2020; Esteves *et al.,* 2021). In 2017, Agarwal *et al*. (2017) gave specific guidelines for SDF testing which was endorsed by the Society for Translational Medicine. This guideline recommended SDF testing in patients with unexplained infertility, varicocele, RPL, unexplained IUI, IVF and ICSI failure cases and in patients exposed to lifestyle risk factors and environmental toxicants.

### 1. Varicocele

Varicocele and SDF are correlated in both fertile and infertile men. Varicocelectomy reduced the level of SDF in males and was observed to increase pregnancy rates (Malhotra, 2017). Oxidative stress and venous stasis were found to be the associated reasons for high SDF and testicular dysfunction in varicocele males (Wright *et al*., 2014). Prominent SDF levels have been shown in males with high-grade varicoceles (grades 2 and 3), however, its association with males with low-grade varicoceles has not been associated yet (Malhotra, 2017). A varicocelectomy decision could be made using SDF tests for reference. Varicocelectomy can be preferred in patients having varicocele and marginal semen parameters (González-Marín *et al*., 2012). Varicocele grade 2 and 3 males with standard semen parameters can undergo SDF testing while this test completely supports varicocele grade 1 having abnormal or borderline semen standard (Cho *et al*., 2017).

### 2. Assisted Reproductive Technique Failures [IVF/ICSI]

We have already mentioned the relation between SDF and ART (IVF/ICSI) when it comes to pregnancy rates. Both pregnancy outcomes and male partner SDF levels are related directly (Zhao *et al*., 2014). Although the effect of SDF on pregnancy rate was reported by many researchers during IVF study, these studies also had their limitations (Osman *et al*., 2015).

It has been seen that ejaculated sperm and testicular sperm have differences in their SDF levels and testicular sperms are being the better ones with lower SDF levels (Moskovtsev *et al*., 2010). Using testicular sperms in ICSI has promoted more success (Pabuccu *et al*., 2017). This research gained the importance of testicular sperm usage in ICSI to overcome previous fertilisation failure of males with high levels of SDF and oligozoospermia. Usage of SDF testing may predict the result of an ART cycle done in infertile males. To overcome ART failures, different approaches of oral antioxidants, sperm selection and frequent ejaculation should be approached to reduce high SDF impact and to get a better result of ART.

### 3. Intrauterine Insemination (IUI) Failures/ Unexplained Infertility/ Recurrent Pregnancy Loss

Patients having undergone IUI were predicted to have decreased pregnancy and delivery rates when SDF index by SCSA was >30% (Wright *et al*., 2014). Another study described IUI outcomes can be determined by two main factors like age and SDF where >12% of SDF resulted in no pregnancy (Agarwal *et al*., 2020). More than 27% SDF during sperm chromatin structure assay (SCSA) showed a negative effect on pregnancy rate during IUI (Rilcheva *et al*., 2016).

Evidence was put forward supporting the relation between SDF level and poor reproductive outcomes during natural conception and intrauterine insemination (Cho & Agarwal, 2017). Following IVF and ICSI, high SDF was found to be a major cause of rise in pregnancy loss (Rilcheva *et al*., 2016). Which was supported in a study of 2969 couples where 2.16 fold increase in pregnancy loss in semen having high SDF during IVF and ICSI (Robinson *et al*., 2012). Twenty four couples were screened having unexplained RPL for SDF level compared to donors of known fertility & general unscreened population of men, where increase in SDF in RPL as compared to general population (Agarwal *et al*., 2020).

## TREATMENT AND PREVENTION OF SDF

Males with normozoospermic sperm parameters but increased SDF levels are associated with the rise in failures of IVF and ICSI procedures. DNA integrity can be altered by different factors including biological, environmental, physical and chemical. Focussing majorly on changing or improving lifestyle can aid to improve sperm quality (Wright *et al*., 2014). SDF is a major concern in male infertility thus more emphasis is to be place on its treatment and prevention (Table 2).

**Table 2:** Treatment and preventive measures for SDF.

Treatment	Process	References
Improving Lifestyle	Maintaining BMI, reducing obesity, Breaking smoking and drinking habit, Improving sleeping habit and stress management either through exercise, weight management, bariatric surgery, behavioral cognitive therapy	Wood *et al*., 2020 Humaidan *et al*., 2022
Antioxidant therapy	Use of vit A, C, E, other micronutrient (L-carnitine, N-acetyl cysteine) and elements (coenzyme Q, zinc, selenium, folic acid) reduced the DFI level in male patients as well as enhance the pregnancy rate in infertile couple.	Wright *et al*., 2014 Alahmar *et al*., 2021 Humaidan *et al*., 2022
Varicocelectomy	Varicocelectomy reduced the SDF level by 4% and increased the chance of natural conception.	Fathi *et al*., 2021 Lira Neto *et al*., 2021
Short abstinence	Reducing the abstinence period to 1 day positively affected the DFI level due to less exposure to oxidative stress.	Agarwal *et al*., 2016b Esteves *et al*., 2015b
Testicular sperm	Many of the cases have significantly reduced DFI and gave high success rate on using testicular sperm in ART and infertile men	Esteves *et al*., 2015b Mehta *et al*., 2015
Sperm processing and preparation	Use of magnet activated cell sorting and physiological ICSI in selecting sperm during ART helped in removing sperm with SDF	Pacheco *et al*., 2020

**Figure 4:**
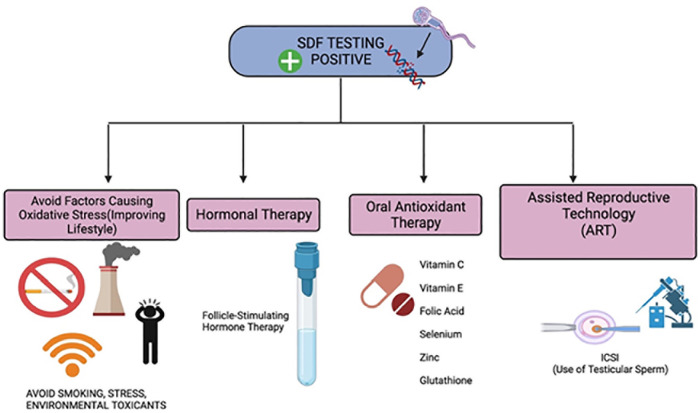
Depending on the severity of SDF, treatment can be done.

Treatment like varicocele repair, hormonal therapy using Follicle Stimulating Hormone (FSH), antioxidant therapy, lifestyle changes and use of testicular spermatozoa in ICSI for males with low sperm counts are few ways to decrease SDF levels and increase pregnancy chance (Esteves *et al*., 2020) (Figure 4), are elaborated in the section below:

### a) Improving lifestyle

Body Mass Index (BMI) along with metabolic syndrome (MetS) was involved in affecting SDF level specifically for overweight individuals. Males with high BMI and associated MetS should be evaluated for SDF testing (Le *et al*., 2019). Obesity results in hormonal shifts, where LH, FSH, and estradiol are linked with higher levels and testosterone with lower levels. Bariatric Surgery can be useful in these cases as it helps in balancing reproductive hormones and SDF levels (Wood *et al*., 2020). Nutraceutical having myo-inositol, alpha lipoic acid, coenzyme Q10, Selenium, zinc and vit B may affect idiopathic infertile men thus reducing their SDF level along with improving semen parameter and vitality of sperm (Delbarba *et al*., 2020) (Table 3). Break of smoking and drinking habits along with indulging in exercise and weight management can be done to improve SDF levels (Kim, 2018). Like high BMI males were subjected to weight loss which was shown to improve DFI (Wood *et al*., 2020). Stress which is one of the causative factors for rise in SDF can be reduced through behavioural cognitive therapy, mind-body psychological stress management practice, meditation and behavioural therapy that can overall manage stress (Bhongade *et al*., 2015). Sleeping cycle and ample amount of sleep is also required to maintain semen quality (Viganò *et al*., 2017). Three months lifestyle intervention program which included diet and exercise was done on patients having DFI>15%. Reduced median DFI from 25.8% to 18% was seen after intervention (Humaidan *et al.,* 2022).

**Table 3:** Treatment that can be adapted from different dietary sources which act as antioxidants which might control high SDF levels (Wright et al., 2014).

Vitamin C	Vitamin E	Zinc	Selenium
Papaya Bell peppers Strawberries Broccoli Pineapple Kiwi Oranges Cantaloupe Kale Cauliflower	Spinach Swiss chard Sunflower seeds Almonds Asparagus Bell peppers Cayenne Pepper Papaya Kale	Spinach Shiitake mushroom Cremini mushroom Organic Lamb Organic Beef Scallops Sesame Seeds Pumpkin seeds Oats	Halibut Tuna Cod Shrimp Cremini mushroom Mustard seeds Sardines Salmon Turkey barley

### b) Antioxidant therapy

The use of the antioxidant therapy in improving SDF levels is still debatable, where some reports have shown a positive prognosis while other studies did not show any usefulness. High ROS levels result in damage to nuclear and mitochondrial DNA, causing base modification, strand break, and cross link chromatin (Cho & Agarwal, 2017). Also, ROS imbalance increases SDF levels and oral antioxidant therapy can also be used to treat males with increased SDF levels (Wood *et al*., 2020). Antioxidant therapies widely used to control sperm DNA fragmentation, includes the use of vitamin A, C, and E along with micronutrients like L-carnitine, N-acetyl cysteine and elements like zinc and selenium are used (Wright *et al*., 2014)(Table 3).

In one such study, where an antioxidant Menevit (containing zinc, folic acid, vit C, E and selenium) was used in patients with high SDF levels was found to enhance pregnancy rate during IVF-ICSI treatment (Wright *et al*., 2014). It was also found the use of oral antioxidant therapy like Vit C & E to reduce the percentage of DFI in ejaculated spermatozoa for identifying the fall in SDF level, however in another study oral antioxidant therapy was used to enhance the pregnancy and clinical implantation rate in ICSI treatment where sperm DNA damage was found to be a hurdle (Wright *et al*., 2014).

CoenzymeQ are found to have antioxidant properties and can be used to treat males with high SDF levels. In one such report by Alahmar *et al.* (2021), CoQ was used and their effect on semen parameter, SDF and oxidative stress markers were analyzed in oligoasthenozoospermia patients, and was found to have a positive effect on decreasing sperm damage. In another study, pregnancy and clinical implantation rates were reported to improve with a fall in SDF level to normal (Wright *et al*., 2014). Three months of oral anti-oxidant therapy having multivitamins, coenzyme Q10, omega-3 and oligo-elements was done decreasing mean DFI to 7.2% in 31 participated patients having DFI>15% (Humaidan *et al*., 2022). Some of the common dietary sources which can be used as antioxidants are given in Table 3.

### c) Varicocelectomy

High levels of SDF are also found in Varicocele males, and varicocele repair can reduce the levels as low as 3.37%, hence, one of the promising methods for SDF reduction in varicocele males (Wang *et al*., 2012). Another treatment known as varicocelectomy is also found to be useful in SDF reduction. A study conducted post-varicocelectomy showed reduction in SDF levels from 35.2% to 30.2% resulting in 37% of patients conceived naturally and another 24% by ART (Smit *et al*., 2013), suggesting varicocelectomy can help in controlling SDF level and promote pregnancy rate. Reduced SDF levels along with increased pregnancy rates were found post 1-year of varicocelectomy treatment, resulting in an overall 62% patients conceiving spontaneously with SDF level less than 25% (Fathi *et al.,* 2021). The Meta-analysis on SDF rate before and after varicocelectomy was done where weighted mean difference (WMD) with 95% confidence level was reported. Reduced postoperative SDF with WMD -7.23% was found. Treatment was more effective in men with high level as compared to normal preoperative SDF level (Lira Neto *et al*., 2021).

### d) Short abstinence

Shorter abstinence and SDF levels are interdependent. A 22.25% DNA damage was found to be improved when the abstinence period was around 1-gap should be there between 2 and days (Agarwal *et al*., 2016b; Gosálvez *et al*., 2011). The concept behind this is that sperms get shorter exposure to oxidative stress in shorter abstinence during its travel through epididymis resulting in lower SDF levels (Esteves *et al*., 2015b). In another study where males with high DFI in their semen were studied by reducing the abstinence period to one day, 91.4% males showed low SDF levels (<30%) (Pons *et al*., 2013).

### e) Use of testicular sperm for intracytoplasmic sperm injection

It has been found that testicular spermatozoa have much better DNA quality than ejaculated sperm (3-5-fold). the use of these testicular sperm showed a positive rise in ICSI in oligozoospermic with high DFI levels (Esteves *et al*., 2017; Pabuccu *et al*., 2017; Alharbi *et al*., 2020). A positive effect was seen on live birth rates using testicular sperms in 172 idiopathic oligozoospermia infertile males having high DFI from 26.4% to 46.7% (Esteves *et al*., 2015a).

### f) Sperm processing and selection media and devices

During ART, sperms of males with high SDF levels can be isolated by different procedures of sperm selection including density gradient centrifugation, intracytoplasmic morphological sperm selection (IMSI), electrophoretic isolation, physiological ICSI (PICSI) dishes, hyaluronic acid binding assay like Sperm-Slow and magnetic cell sorting (MACS) (Degheidy *et al*., 2015; Punjabi *et al*., 2018).

Although MACS, PICSI and IMSI used in IVF techniques along with ICSI have not shown much better results (Rappa *et al*., 2016), however, an increase in 28.75% in IMSI and 38.3% in PICSI rise was observed in birth rates compared to 24.2% in normal ICSI (Bradley *et al*., 2016). Impact of density gradient centrifugation and swim up technique in ART for sperm preparation is not yet clear (Nadalini *et al*., 2014) and MACS can be used to have a good quality of semen by removing apoptotic ones having higher levels of SDF and can lead to improved fertility outcomes (Pacheco *et al*., 2020). During PICSI, the use of Hyaluronic acid can help to select spermatozoa having normal nuclei with no SDF, thus helping at the time of ICSI (Parmegiani *et al*., 2010).

## CONCLUSION AND FUTURE PROSPECTIVE

For normal fertilization, selection of spermatozoa with high DNA integrity is very essential, and to improve the fertilization rates various sperm selection techniques are being used worldwide. Recent advancements in technology and fertility research have resulted in development more accurate and efficient SDF tests to increase awareness about male factor infertility. Due to the increase in the role of male factor infertility, it is very important to focus on advanced causes of infertility apart from the traditional semen analysis. DNA damage in couples should be found prior to any ART treatment, especially in unexplained infertility couples and males with normal sperm count. Finding DNA fragmentation levels will help in managing infertility issues in a much better way among couples and can also improve ART outcomes. Inability to adapt SDF testing on a regular basis in infertility clinics may create a barrier to managing proper line of treatment. Recent advanced sperm selection techniques can be employed in couples with male infertility, which can improve sperm selection and ultimately clinical outcomes.

The SDF analysis should be used as a diagnostic tool to help manage a couple’s infertility treatment. Patients presenting with the following should have a DNA fragmentation test:

- All idiopathic couples: 40-50% of men having idiopathic infertility have higher sperm DNA fragmentation. The approach to treat this high DFI in idiopathic male infertility can be by use of testicular sperm while performing ART or use of nutraceuticals like vit B, Coenzyme Q, myoinositol and
- Men older than 40 years, even if prior fertility: Increase in age is often related to increase in DFI, which can be due to increased oxidative stress. Comet assay can be used to identify the status of such infertility in men which can further help in selecting spermatozoa with the help of MACS. However, treatment can be done with the help of ART along with the use of antioxidant therapy.
- Men with known exposures to toxicants: People that have been exposed to radiotherapy, chemotherapy, tobacco, smoke usually have the problem of infertility due to oxidative stress. Their semen should be visualized for SDF with the help of comet or SCD and then treatment should be decided. Antioxidant therapy, use of testicular sperm, improving lifestyle with adaption of ART are some important prospective to reduce the SDF and its related infertility.
